# Modeling Alzheimer’s disease in transgenic rats

**DOI:** 10.1186/1750-1326-8-37

**Published:** 2013-10-25

**Authors:** Sonia Do Carmo, A Claudio Cuello

**Affiliations:** 1 Department of Pharmacology and Therapeutics, McGill University, 3655 Promenade Sir-William-Osler, Room 1325, Montreal, Quebec H3G 1Y6, Canada; 2 Department of Anatomy and Cell Biology, McGill University, Montreal, Quebec H3A 2B2, Canada; 3 Department of Neurology and Neurosurgery, McGill University, Montreal, Quebec H3A 2B4, Canada

**Keywords:** Alzheimer’s disease, Rat, Transgenic, Behavior, Early pathology, Amyloid, Intracellular, Mature plaques, Tau, Neuronal loss

## Abstract

Alzheimer’s disease (AD) is the most common form of dementia. At the diagnostic stage, the AD brain is characterized by the accumulation of extracellular amyloid plaques, intracellular neurofibrillary tangles and neuronal loss. Despite the large variety of therapeutic approaches, this condition remains incurable, since at the time of clinical diagnosis, the brain has already suffered irreversible and extensive damage. In recent years, it has become evident that AD starts decades prior to its clinical presentation. In this regard, transgenic animal models can shed much light on the mechanisms underlying this “pre-clinical” stage, enabling the identification and validation of new therapeutic targets. This paper summarizes the formidable efforts to create models mimicking the various aspects of AD pathology in the rat. Transgenic rat models offer distinctive advantages over mice. Rats are physiologically, genetically and morphologically closer to humans. More importantly, the rat has a well-characterized, rich behavioral display. Consequently, rat models of AD should allow a more sophisticated and accurate assessment of the impact of pathology and novel therapeutics on cognitive outcomes.

## Introduction

Despite important advances in our understanding of the molecular basis of Alzheimer’s disease (AD) neuropathology and the large variety of novel therapeutic approaches attempted, this condition remains incurable. The recent failure of passive immunotherapy clinical trials (Bapineuzumab and Solanezeumab) best illustrates the challenges that lay ahead [1]. It seems that the main obstacle to an effective therapy is that, at the time of clinical diagnosis, the brain has already suffered extensive and perhaps irreparable damage.

At the diagnostic stage, the AD brain is characterized by abundant senile amyloid plaques, formed by extracellular aggregates of amyloid-beta (Aβ) peptides, and by neurofibrillary tangles (NFTs) consisting of intracellular aggregates of abnormally phosphorylated tau (p-tau) protein (reviewed in [2-4]). The neurodegenerative changes in the central nervous system (CNS) neurons and synapses ultimately lead to the progressive cognitive decline characteristic of AD [5].

There is also substantive evidence indicating that CNS pro-inflammatory mechanisms contribute to cognitive impairment [6,7]. Inflammatory processes in AD have been classically regarded as an Aβ plaque-induced event, characterized by activation of microglia and astrocytes surrounding the plaques [8,9].

In recent years, it has become evident that AD starts decades prior to its clinical presentation, based on biomarker studies in familial and sporadic forms of the disease [10-12]. Exploring the neuropathology of AD in such human “pre-clinical” stages is not an easy task. Transgenic animal models can shed much light on the many factors impacting or accelerating disease progression. They can also be of value to identify new biomarkers or potential new therapeutic targets. Transgenic rat models of the AD-like pathology offer distinctive advantages, as will be discussed below.

### Transgenic models of AD

Our knowledge of the molecular mechanisms underlying AD pathogenesis has made a leap forward with the creation of the first transgenic AD mouse models in the mid 1990s [13-15]. Based on the identification of the mutations in the amyloid precursor protein (APP), presenilin 1 (PS1) and presenilin 2 (PS2) genes involved in the familial forms of AD, these models reflect various aspects of AD including amyloid accumulation, abnormal tau phosphorylation, inflammation or behavioral deficits [13-17].

Transgenic mouse models have made an important contribution to testing the so-called “Amyloid Hypothesis”. Furthermore, they have played a role in assessing a large variety of disease-modifying compounds. Given the progressive and predictable evolution of the pathology, the use of transgenic models also offers the opportunity to find reliable biomarkers, crucial in detecting the disease at the early, “silent”, asymptomatic stage, during which therapeutic interventions would have much greater possibilities of success.

Although transgenic mouse models have proven highly valuable in elucidating the mechanisms involved in AD, the recent failure of AD immunotherapy highlights the necessity of developing superior models of the AD pathology. The ideal transgenic model should mimic multiple aspects of the disease including its etiology and a time-dependent progression of the pathology, involving similar structures and cells similar to the human pathology. Most importantly, however, the model should provide reliable, longitudinal readings about the status of higher brain function by applying suitable learning and memory tests, analysis of body fluids, such as cerebrospinal fluid, and imaging. Such models should be free of confounding factors such as impairments in visual or motor systems, which can be falsely interpreted as AD deficits provoked by nonspecific transgene- or strain-effects.

### Advantages of using rats: the rat as a model for AD

Historically, mice were preferred over rats for transgenesis mainly due to technical reasons. Compared to mice, rat one-cell embryos have less visible pronuclei and more flexible plasma and pronuclear membranes, making transgene injection in pronuclei more difficult [18]. The low survival of embryos following injection also contributes to making rat transgenesis more demanding and time-consuming [18]. Additionally, tools for manipulation of the rat genome are less readily available [19]. Until recently, embryonic stem (ES) cell-based targeting technology, a powerful tool allowing gene replacement (knock-in) or loss of function mutations (knock-out), was not available, as viable rat ES cells had been difficult to obtain [20].

Despite these drawbacks, rats offer numerous advantages compared to mice. The rat is physiologically, genetically and morphologically closer to humans than mice [21-23]. Its larger body and brain size facilitates intrathecal administration of drugs, microdialysis, multiple sampling of cerebrospinal fluid, *in vivo* electrophysiology, as well as neurosurgical and neuroimaging procedures [19]. Of relevance for AD modeling, similarly to humans, the rat contains 6 isoforms of tau [24], although the ratio of 4R/3R tau isoforms is different (9:1 in rats; 1:1 in humans). In addition, there is good homology between the rat and human apoE amino acid sequences (73.5% with human apoE3, 73.9% with apoE4) [25,26]. However, while its sequence is more similar to apoE4, rat apoE displays the biophysical behavior of apoE3 [27].

Another major advantage of this species is that it is behaviorally well characterized. Rats have finer and more accurate motor coordination than mice and exhibit a richer behavioral display. They also display a more complex social behavior. They display juvenile play fighting and courtship as well as low levels of aggression [28]. Since the rat is a terrestrial, aquatic and arboreal mammal, it is more competent and less stressed in water-based navigation tasks such as the Morris water maze (MWM) [28]. These behavioral differences may be accounted for by the fact that rats, like humans, and opposed to mice, have a post-natal brain development that would lead to a greater number of synapses and a more complex synaptic organization [28]. Consequently, rat models of AD should allow a more sophisticated characterization at the behavioral level and thus enable a more accurate assessment of the impact of the pathology on cognitive outcomes. They should also enable a better assessment of the effects of potential therapeutics on cognition in longitudinal studies.

Based on these advantages, rats are increasingly and successfully used to mimic key pathological hallmarks of neurodegenerative diseases including Alzheimer’s (as discussed in this review), Parkinson’s (PD) [29-31], Huntington’s (HD) [32], amyotrophic lateral sclerosis [33] and tauopathies [34,35].

Importantly, it has been reported that some transgenic rat models offer a more accurate representation of the human disease compared to mice bearing the same transgene. This has been exemplified in hypertension [36] and atherosclerosis [37], as well as in models of neurodegenerative diseases. Thus, mouse models of HD can only mimic juvenile HD pathological changes whereas HD transgenic rats allow study of the common adult type of the disease [32]. Also, no significant loss of dopaminergic neurons is observed in the human alpha-synuclein transgenic mouse model of PD, but severe loss of the dopaminergic integrity is reported in human alpha-synuclein transgenic rats [31].

### Early rat models of AD

Rats have played a prominent role in the modeling of AD, well before the advent of transgenesis. However, most of the models summarized in this section do not represent accurate model systems for AD as they do not exhibit neuritic plaques, NFTs or neuron loss. This is the case, for example, in aged rats, which reflect only some aspects of human aging, such as learning and memory impairments and moderate deficits in cortical cholinergic and dopaminergic function [38-40].

Chemical and lesion-induced rat models have been extensively used, particularly to test the cholinergic hypothesis of AD. This hypothesis states that CNS cholinergic deficits in elderly adults and demented patients are the main factors responsible for their cognitive impairments [41-43], and has led to the well-established, symptomatic, anticholinesterase therapies (for review see [44]).

A large variety of compounds have been used to induce AD-like cortical cholinergic neuronal loss with varying degrees of specificity. These include the relatively non-specific scopolamine [45,46] and the p75NTR-specific immunotoxin for cholinergic neurons, 192-IgG-saporin [47,48]. Several rat lesion models have been used, and include models of brain trauma [49], bilateral transection of the hippocampal fimbria-fornix [50], ovariectomy [51] and hyperthermia on the post-ischemic brain [52], the last creating AD-like pathology [52].

The finding that Aβ is central to the development of plaques [53] and is neurotoxic [54] has led to studies investigating the impact of Aβ on brain function *in vivo*. However, although cerebral infusion of Aβ in naïve rats can recapitulate some key features of human AD including cholinergic dysfunction, Aβ deposits, ventricular enlargement, neuron loss and behavior deficits, it can not simulate the progressive neurodegeneration characteristic of AD [55-58]. In addition, there is great inconsistency between Aβ infusion models, likely due to differences in methodology such as identity, type (fresh, presumably oligomeric, versus fibrillar forms) and the concentration of peptide administered, duration of the treatment and site of infusion. Therefore, while such models might be of value to examine specific aspects of the amyloid pathology, they fail to reproduce the full spectrum of AD neuropathological hallmarks.

### Virus-mediated rat models of AD

More recently, the advance of virus-mediated gene transfer technology has allowed for the expression of human APP (hAPP)695 bearing the Swedish mutation in the adult rat hippocampus. This induced Aβ42 immunoreactivity and learning deficits in the MWM up to 12 months post-injection. However, the injection did not lead to Aβ plaque deposition, gliosis or neural loss [59]. Virally-mediated gene transfer of human Aβ42 and Aβ40 peptides bearing the British mutation into the hippocampus of adult Wistar rats has also helped to establish the contribution of each Aβ species in AD [60].

### Transgenic rat models of AD

Since the early 2000s, a wide array of transgenic rats has been created based on the expression of human genes relevant for early-onset familial AD such as wild-type or mutated APP and mutated PS1. These models offer a large heterogeneity in their phenotype, which arises from several factors. First, the expression of these transgenes is controlled by different neuronal promoters resulting in varying expression strengths and patterns. In addition, these models were produced and stabilized in both inbred (Lewis and Fischer-344) or outbred (Sprague Dawley and Wistar) genetic backgrounds [18]. It is now well established that genetic background has a great influence on pathogenesis. For example, it was demonstrated that rat strain SHR72 expressing human truncated tau (151–391, 4R) under the control of the Thy1 promoter (Table 1) displayed different NFT load and neuroinflammation markers depending on the background in which it was stabilized [61]. Differences in expression strength can also result from the method used to introduce the transgene in fertilized eggs (pronuclear injection of DNA or lentiviral delivery). Most importantly, differences in phenotypes may be explained by the introduction of single, double or triple transgenes.

**Table 1:** Transgenic rat models of Alzheimer’s disease

**Name**	**Transgene**	**Background**	**Amyloid pathology**	**Tau pathology**	**Other**	**References**
UKUR28	hAPP751 Swe, Ind PDGF promoter	Wistar (outbred)	iAβ in cortex and hippocampus from 6 mo No plaques	N/A	Increased pERK2	[62,63]
UKUR25	hAPP751 Swe, Ind Human PS1 (M146L) PDGF promoter	Wistar (outbred)	iAβ in cortex and hippocampus from 6 mo No plaques	Increased ptau (PHF-1) at 9 mo, no tangles	Increased pERK2, decreased p- p90RSK Altered proteome (SELDI-TOFF MS) Altered subcellular compartments	[62-65]
TgAPPswe	hAPP751 Swe, PDGF promoter	Fisher-344 (inbred)	Increased APP mRNA (56%) and Aβ-40 and Aβ-42 peptides, no plaques	N/A	Better cognitive performance in MWM and STFP	[68]
Tg6590	hAPP695 Swe, UbiquitinC promoter	Sprague-Dawley (outbred)	Increased APP products Cerebrovascular deposits at 15 mo Few diffuse plaques	Increased ptau (PHF-1) at 15 mo, no tangles.	Impairment in MWM and open-field	[75,76]
hAPP695	hAPP695 wild-type, UbiquitinC promoter	Wistar (outbred)	Increased APP/ Aβ levels (2 fold) in cortex and hippocampus no plaques	N/A	Smaller infarct volume impairment in MWM and BWT after MCAO	[72]
APP21 APP31	hAPP695 Swe, Ind, UbiquitinC promoter Lentiviral delivery	Fisher-344 (inbred)	Increased APP products and APP mRNA in brain (2.9 fold), kidneys and lungs Increased Aβ-40 and Aβ-42 in serum No plaques	N/A		[73,74]
PSAPP Tg478/ Tg1116/ Tg11587	hAPP695 Swe, Rat synapsin I promoter hAPP695 Swe, Lon, PDGFβ promoter Human PS1 (M146V), Rat synapsin I promoter	Sprague-Dawley (inbred)	Mostly diffuse plaques Few compact plaques in hippocampus. No vascular Aβ deposits Aβ load confirmed with (F-18) FDDNP microPET	Increased ptau (AT8, PHF-1), no tangles	Impairment in LTP and in MWM performance Activation of astrocytes and few microglia particularly around plaques No neuronal loss	[77-79]
McGill-R-Thy1-APP	hAPP751 Swe, Ind, Mouse Thy1.2 promoter	Wistar (outbred)	Progressive accumulation of iAβ in cortex and hippocampus from 1 week post-natal Aβ plaques starting at 6-9 mo	N/A	Dystrophic neurites and astrogliosis around plaques Progressive learning deficits (MWM) Altered metabolites (MRS)	[81,85]
TgF344-AD	hAPP695 Swe, Human PS1ΔE9 Mouse PrP promoter	Fisher-344 (inbred)	Progressive accumulation of iAβ, Aβ-40 and Aβ-42 and Aβ plaques	Increased ptau (CP-13, pTau-PADRE and others) and Gallyas-positive NFT	Deficits in open-field, NOR, BM Presence of dystrophic neurites, activated astrocytes and microglia around plaques. Neuronal loss	[91]
SHR72 and SHR318	Human tau truncated (151-391, 4R) Mouse Thy1 promoter	SHR (inbred)	N/A	Increased ptau (AT8) Tangles in brainstem	Deficits in MWM and BWT Impaired reflex responses no neuronal loss in brain, axonal damage in the brain stem and spinal cord, decreased lifespan	[35,95,96]
SHR24	Human tau truncated (151-391, 3R) Mouse Thy1 promoter	SHR (inbred)	N/A	Increased ptau (DC11 and others) Tangles in cortex	No neuronal loss in cortex and hippocampus, decreased lifespan	[97]

*BM* Barnes maze, *BWT* beam walking test, *IHC* immunohistochemistry, *MCAO* middle cerebral artery occlusion, *mo* months-old, *MRS* magnetic resonance spectroscopy, *MWM* Morris water maze, *N*/*A* information not available, *NFT* neurofibrillary tangles, *NOR* novel object recognition, *PET* positron emission tomography, *STFP* social transmission of food preference.

### Rat models of amyloid pathology devoid of plaques

The earliest transgenic rat models of AD showed accumulation of intracellular Aβ (iAβ) but no senile plaques. It was suggested that this was due to inadequate Aβ levels, since higher concentrations are required to initiate the deposition process. Some of these models however, did display synaptic dysfunction (LTP and behavior) supporting the view that cognitive deficits are independent of plaque formation but correlate better with Aβ oligomers and other Aβ species.

Our first attempts to generate rat models of AD failed to reproduce the classical AD pathological hallmarks (Table 1) [62-65]. This can be attributed to moderate levels of APP gene expression. However, UKUR25 and UKUR28 transgenic rat strains showed an important accumulation of intracellular Aβ (iAβ)-immunoreactive material in pyramidal neurons of the neocortex and in CA2 and CA3 regions of the hippocampus. These models significantly contributed in supporting the role of iAβ in the amyloid cascade at the early, pre-plaque phase of the amyloid pathology. Indeed, they confirmed *in vivo* that the accumulation of iAβ material, in the absence of plaques, induces deregulated ERK2 activation [62,63] as previously demonstrated *in vitro*[66,67]. Furthermore, they also demonstrate that iAβ is sufficient to trigger the initial steps of the tau-phosphorylation cascade, learning impairments in the MWM task [62,63], significant changes in the hippocampal proteome, particularly in synaptic proteins implicated in learning and memory formation [65] and morphological alterations in the Golgi apparatus, lysosomes and lipofuscin bodies [64].

Intracellular iAβ accumulation was observed in other rat models of amyloid pathology. Similarly to our model, TgAPPswe rats do not develop extracellular plaques or NFTs up to 18 months of age [68]. These rats show a mild increase in APP mRNA (56.8% at 12 months). In contrast with UKUR25, these rats perform better at 6 and 12 months old in two hippocampus-dependent tasks, the MWM and the social transmission of food preference task when compared with non-transgenic animals. This discrepancy can be explained by differences in the genetic background and the transgene expressed (Table 1), as TgAPPswe rats do not carry the APP Indiana and the PS1 Finn mutations, which could result in lower iAβ levels. Accordingly, the authors suggest a dose-dependent effect of APP, which would play a role in normal learning and memory processes at low doses but would lead to neurodegeneration and cognitive decline at higher doses [68].

Models expressing wild-type or mutated hAPP isoform 695 (hAPP695) have also been attempted. This choice is justified, as it is regarded as the isoform preferentially expressed by neurons. In addition, it was reported that there is a selective loss of APP695 transcripts in the brain of AD-affected patients [69]. However, it has been shown *in vitro* and in transgenic mouse models that overexpression of hAPP751 causes more AD-like pathology and cognitive impairments than hAPP695 [70,71].

Trangenesis with wild-type hAPP695 results in a two-fold increase of APP/Aβ fragments in cortex and hippocampus compared to non-transgenic animals, leading to behavioral impairments after middle cerebral artery occlusion [72]. Higher levels of APP expression (2.9 fold more APP mRNA) were first achieved in the brain of APP21 and APP31 inbred models which were created by injecting recombinant lentivirus carrying the hAPP695 with the Swedish and Indiana mutations into zygotes (Table 1) [73]. These models have substantial quantities of Aβ40 and 42 in serum, and especially so in homozygous animals. Circulating Aβ most likely does not arise from the brain, as the models also express high levels of APP mRNA in the kidneys, heart and lungs. Desspite of the high levels of human APP in neurons of the cortex and hippocampus, they do not develop extracellular deposits of Aβ [74]. However, senile plaques and cerebral Aβ angiopathy can be observed 9 months after the cerebral injection of dilute brain extracts from AD patients into APP21 animals, suggesting that Aβ deposition can be exogenously seeded if the host expresses human Aβ [74].

A third rat model expressing hAPP695 with the Swedish mutation (Table 1) has shown an accumulation of iAβ in neurons of the cortex, hippocampus and cerebellum and an increased amount of soluble Aβ material. At 9 months old, Tg6590 exhibits impaired spatial learning in the MWM and altered spontaneous activity in the open-field [75]. In addition, magnetic resonance imaging (MRI) suggests a tendency towards enlargement of the lateral ventricles at 11 months old [75]. At 15 months of age, these rats show Aβ cerebrovascular deposits, rare diffuse plaques, and tau hyperphosphorylation at PHF-1 site without the formation of mature plaques or NFTs even by the age of 22 months [75,76].

### Rat models of amyloid pathology with mature plaques

The first model to develop amyloid plaques was achieved in homozygous double transgenic rats Tg478/Tg1116 expressing hAPP695 carrying the Swedish and Swedish/London mutations. These rats show increased APP, Aβ40 and Aβ42 load and developed diffuse plaques by 17–18 months of age [77]. The age of plaque onset was accelerated to 9 months by introducing a third transgene carrying a human mutated presenilin gene [77,78]. From the age of 9 months-old, PSAPP rats (also named Tg478/Tg1116/Tg11587) display abundant diffuse plaques in the cortex, hippocampus, olfactory bulb, thalamus and hypothalamus but not in the cerebellum or brain-stem (Table 1). However, only few compact plaques are detectable in the hippocampus even at 22 months-old and no vascular Aβ deposits are observed. The progressive accumulation of Aβ plaques was confirmed with the use of [F-18]FDDNP micro positron emission tomography [79]. Astrocytic and light microglial activation and tau hyperphosphorylation is present around compact plaques. These rats also show impaired LTP accompanied by progressive behavior deficits in the MWM task, detectable at 7 months of age, in the absence of plaques. Behavior deficits correlate with Aβ42 load in the hippocampus. However, these rats lack neurofibrillary pathology or neuronal loss [78]. Its use as an efficient AD model is also hampered by a tendency towards premature death related to kidney disease, hypertension and immunosuppression, which are likely a consequence of the genetic disturbance caused by the presence of the triple transgenes [80].

**Figure 1:**
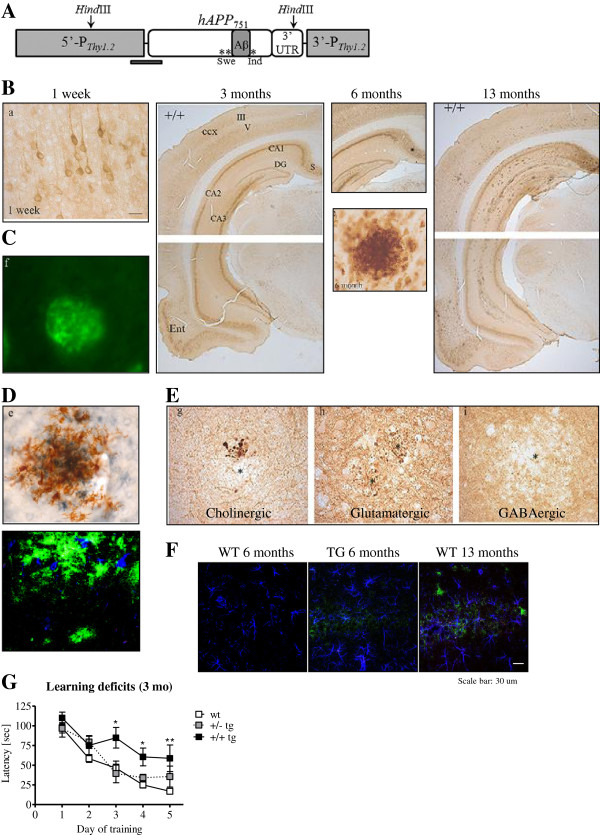
**The McGill-R-Thy1-APP transgenic rat phenotype. (A)** The McGill-R-Thy1-APP transgenic rat expresses the human APP751, bearing the Swedish and Indiana mutations under the control of the murine Thy1.2 promoter. Its phenotype is fairly similar to the human pathology reported in AD and MCI. **(B)** We observe intraneuronal Aβ accumulation starting at 1 week post-natal, as determined with our murine monoclonal antibody (McSA1) against the N-terminus of the Aβ peptide. The development of plaques follows the same anatomical sequence as in humans. Mature amyloid plaques are Thioflavin S-positive **(C)** and are surrounded by activated microglia as observed with MHCII- (brown) and Aβ-specific antibodies (McSA1-blue) and also with Iba-1(blue) and McSA1(blue) **(D)**. Plaques are also accompanied by dystrophic neurites **(E)** and astrogliosis (GFAP-blue, McSA1-green) **(F)**. **(G)** These rats already show learning deficits in the Morris water maze task at the pre-plaque stage (3 months old) and these deficits progress with amyloid accumulation. Images adapted from [81] with the publisher’s permission and from [83].

The McGill-R-Thy1-APP rat model is the only model able to reproduce extensive AD-like amyloid pathology with a single transgene (Figure 1) [81]. This model expresses the hAPP751, bearing the Swedish and Indiana mutations under the control of the murine Thy1.2 promoter. In the McGill-R-Thy1-APP transgenic rat, a single transgene is able to produce human APP expression specifically in AD-relevant areas of the brain without cerebellar and peripheral tissue expression. The presence of a single transgene with a low copy number makes of this rat the least genetically aggressive AD transgenic model developed so far.

McGill-R-Thy1-APP rats display iAβ as soon as one week post natal in the cortex and hippocampus in both hemi and homozygous animals. The oligomeric nature of the iAβ material was confirmed using the NU-1 monoclonal antibody that specifically recognizes soluble aggregates of Aβ [82]. The pathology is dose-dependent as, in homozygous animals, iAβ accumulation leads to progressive neuritic plaque deposition starting from 6–9 months old. Hemizygous rats develop no or very few plaques at much later stages. The anatomical spreading of plaques coincides with that observed in human AD, starting from the subiculum and expanding to the entorhinal cortex and hippocampus. The AD-like pathological phenotype also includes the presence of transmitter-specific dystrophic neurites (Figure 1) [81]. Moreover, we have recently observed a recruitment of microglia towards iAβ-burdened hippocampal neurons and a remarkable pre-plaque astrogliosis [83]. It is of significance that the McGill-R-Thy1-APP rat presents progressive behavior impairments in the MWM starting at 3 months of age while no amyloid plaques are yet present. The deficits are transgene-dose-dependent and they correlate with the abundance of the 12kDa 6E10-immureactive band likely corresponding to a combination of Aβ trimers and the APP C-terminal fragment, C99 [81]. This observation reinforces the hypothesis on the impact of oligomeric iAβ in cognition [84].

These rats were also used to study metabolite levels by magnetic resonance spectroscopy in the dorsal hippocampus and frontal cortex. The findings demonstrate complex metabolite alterations during the progression of the amyloid pathology, different from those observed during normal aging [85]. MRI imaging on these rats also showed marked brain shrinkage, which is more evident for the hippocampal complex and resting-state connectivity impairment [86-88]. Consistent with these observations, McGill-R-Thy1-APP rats display impairments in firing rates for place discrimination of spatial context [89] and a very compelling *in vivo* impairment in hippocampal LTP formation at preplaque stages [90].

More recently, a bigenic TgF344-AD rat has been reported (Table 1) [91]. These rats express hAPP695, with the Swedish mutation, and PS1ΔE9 under the control of the strong murine PrP promoter. These rats demonstrate strong age-dependent accumulation of iAβ, soluble and insoluble Aβ40 and Aβ42 peptides and thioflavin-positive amyloid plaques. The amyloidosis is associated with hyperactivity in the open-field as well as age-dependent deficits in spatial learning and memory as assessed with the novel object recognition and the Barnes maze tasks. Surprisingly, as it was never seen before in other transgenic rat models of AD and even in transgenic AD mouse models expressing APP and PS1 mutations, by 16 months of age these rats present Gallyas-positive structures resembling NFTs seen in human AD. These structures contain p-tau as detected with several p-tau antibodies. The observed amyloidosis and tauopathy are accompanied by neuronal loss. These rats also present glial activation as early as 6 months old, before appreciable extracellular Aβ deposition [91].

### Rat models of tau pathology

Several transgenic mouse models expressing mutated forms of human tau develop neurofibrillary degeneration [92,93]. Because the rat contains 6 tau isoforms, as do humans, rat models of tau pathology were also created (Table 1) [35,94-97]. Overexpression of human non-mutated truncated tau encompassing 4 repeat domains (151–391, 4R) in neurons leads to a hyperphosphorylation of tau and the development of neurofibrillary degeneration similar to that reported in AD [35]. Behavior analysis highlighted a progressive cognitive decline in spatial navigation in MWM, as well as disturbances in sensorimotor and reflex responses [95]. These impairments correlate with the progressive accumulation of argyrophilic NFTs and mature sarcosyl-insoluble tau complexes and extensive axonal damage in the brain stem and spinal cord. However, although hyperphosphorylated tau was observed in cortex and hippocampus, no neuronal loss or tangles were observed in the brain [95]. These impairments lead to decreased lifespan [35,96]. The first rat model developing progressive NFTs in the cortex expresses a human non-mutated truncated tau encompassing 3 repeat domains (151–391, 3R). These rats develop progressive cortical neurofibrillary degeneration as early as 9 months of age [97]. Surprisingly, this rat does not show neuronal death in the cortex, the region with the largest accumulation of tangles, or the hippocampus, the region presenting the highest expression of human tau. However, the neurofibrillar pathology leads to decreased lifespan. More models of tau transgenesis are likely to appear in coming years.

### Comparison of transgenic rat and mouse models of AD

It has been more complicated to achieve AD-like amyloid deposition in the brain of transgenic rats than mice. The elevation of soluble Aβ or the extent of plaque accumulation is often less in rat (Table 1) than in mouse models expressing similar constructs, resulting in less aggressive phenotypes. Accordingly, Tg6590 [75,76] (Table 1), fail to develop mature plaques despite displaying some cognitive impairments. Conversely, its equivalent in mice, Tg2576, which expresses hAPP695 bearing the Swedish mutations under the control of the PrP promoter, presents cognitive decline accompanied by numerous Aβ plaques [98]. Similarly, TgAPPswe rats show very slight increases in Aβ peptide production with no plaque development [68] while TgAPP23 mice expressing hAPP751 with the Swedish mutations under the control of the Thy-1 promoter show typical plaques by 6 months of age accompanied by neuritic and synaptic degeneration [99]. Furthermore, TgCRND8 mice [100] expressing hAPP695 with the Swedish and Indiana mutations under the control of the PrP promoter develop early and extensive plaque deposition by 3 months of age while APP21 and APP31 rats [73,74] never accumulate extracellular amyloid. In these cases, differences in phenotype might arise from differences in the promoters used in mice and rats. In support to this, TgAPP(Sw,V717F) mice [101] and UKUR28 rats [62,63] expressing hAPP751with the Swedish and Indiana mutations driven by the PDGF promoter have similar phenotypes despite an absence of Aβ42 and plaque accumulation.

However, there are other cases where the exact same construct used in the two species resulted in different phenotypes indicating that species-specific factors likely contribute to these phenotype differences. For example, McGill-Thy1-APP mice [102] and McGill-Thy1-APP rats [81] expressing exactly the same construct containing hAPP751 with the Swedish and Indiana mutations under the control of the Thy1 promoter develop a similar phenotype. However, it is far more aggressive in mice (plaques at 4 months) than in the rat (plaques at 6–9 months), the latter needing to be homozygous to develop plaques. This might be explained by the number of copies of the transgene inserted in the host DNA. While few transgene copies are present in McGill rats [81], McGill mice [102] contain more than 20 copies of the transgene. Phenotypic differences were also observed in TgF344-AD rats [91] and Tg-APPswe/PS1dE9 mice [103,104], both expressing hAPP695 with the Swedish mutations under PrP regulatory control. These models show extensive and progressive amyloid pathology accompanied by cognitive deficits and some tau pathology. However, TgF344-AD rats have greater abundance of soluble oligomeric Aβ species. Also, while TgF344-AD rats display NFT-like structures and frank neuronal loss, these are not observed in Tg-APPswe/PS1dE9 mice.

### Concluding remarks and future directions

Transgenesis in rats offers great potential to decipher subtle and early aspects of AD pathology. The rat is, in many aspects, closer to humans than mice. Because of its predictable and multi-faceted behavioral display, this species is of great value for accurate cognitive assessment. The current rat transgenic models, while replicating all aspects of the amyloid pathology including inflammation and the presence of dystrophic neurites, remain incomplete models as it does not develop “bona fide” human-like neurofibrillary tangles. Despite these weaknesses, current transgenic rat models of AD are contributing significantly to our understanding of processes and mechanisms involved in the disease progression. However, as the available AD rat models display varying degrees of competence in recapitulating the characteristics and severity of the pathology, the choice of the model is crucial for the investigative objectives. Also, although the CNS of rats is closer to humans than that of the mouse, the extrapolation of conclusions from rat to humans would require a measure of caution. These transgenic models are closer to the pathology elicited in familial AD (both genetically driven), however, models with a minimal genetic invasiveness, such as the McGill-R-Thy1-APP, offer the closest available analogy to the human sporadic AD pathology. Despite their obvious limitations, transgenic rat models will be of great assistance in the search of potential biomarkers signaling an early, preclinical, pathology and in the search and validation of novel therapies. From the experimental point of view, they have already shown to be of significant advantage for *in vivo* electrophysiology, imaging, proteomics, epigenetics and in the future for optogenetic studies.

## Abbreviations

AD: Alzheimer’s disease; Aβ: Amyloid-β peptide; APP: Amyloid precursor protein; CNS: Central nervous system; ERK: Extracellular-signal-regulated kinases; HD: Huntington’s disease; MWM: Morris water maze; MRI: Magnetic resonance imaging; NFTs: Neurofibrillary tangles; PD: Parkinson’s disease; PHF: Paired helical filaments; PS1: Presenilin 1; PS2: Presenilin 2.

## Competing interests

The authors declare that they have no competing interests.

## Authors’ contributions

SDC and ACC contributed to the writing and revising of the manuscript. Both authors read and approved the final manuscript.
